# Correlation Between Tumor Volume and Hemoglobin Level in Gastrointestinal Stromal Tumor

**DOI:** 10.7759/cureus.39073

**Published:** 2023-05-16

**Authors:** Yashwant Sakaray, Kishore Abuji, RN Naga Santhosh Irrinki, Naveen Maheshwari, Hemanth Kumar, Lileswar Kaman

**Affiliations:** 1 Department of General Surgery, Postgraduate Institute of Medical Education and Research, Chandigarh, IND

**Keywords:** mesenchymal tumour, haemoglobin, anemia, tumour volume, gist

## Abstract

Introduction: Gastrointestinal stromal tumors (GIST) are the most common mesenchymal tumors. Anemia is a common finding in GIST, but the relationship between tumor volume and anemia severity is not well established.

Methods: This study aimed to investigate the correlation between the severity of anemia and various factors, mainly tumor volume, in GIST patients who underwent surgical resection. The study included 20 patients with GIST who underwent surgical resection at a tertiary care center. Demographic data, clinical presentation, hemoglobin level, radiological findings, surgical procedure, tumor characteristics, pathological findings, and immunohistochemical analysis were recorded. The tumor volume was calculated from the final resected tumor dimensions.

Results: The mean age of the patients was 53.8 ± 12 years. Eleven were males, and nine were females. The most common presentation was upper gastrointestinal bleeding (50%), followed by pain in the abdomen (35%). The most common tumor location was the stomach (75%). The mean hemoglobin level was 10.29 ± 1.9 g/dL. The mean tumor volume was 470.8 ± 1269.07 cc. R0 resection was achieved in 18 (90%) patients. There was no significant correlation between tumor volume and hemoglobin level (r=0.227, p=0.358).

Conclusion: This study found no significant correlation between tumor volume and the severity of anemia in patients with GIST. Further studies with larger sample sizes are needed to validate these findings.

## Introduction

Gastrointestinal stromal tumors (GIST) are the most common mesenchymal tumor, with an incidence of 1%-3% of all gastrointestinal malignancies, accounting for 18% of all sarcomas. GISTs most commonly arise from the stomach, followed by the small intestine, colon and rectum, mesentery or omentum, and esophagus. Rarely arises from the duodenal ampulla, appendix, gall bladder, or urinary bladder. GIST arises from the interstitial cells of Cajal, with the most common mutation being the c-KIT gene in >80% of cases, followed by the platelet-derived growth factor receptor alpha (PDGFRA) gene mutation [1,2].

The most common clinical presentation of GIST is upper gastrointestinal bleeding, including melena and hematemesis, followed by pain in the abdomen, distension, and discomfort due to mass effect. In the published literature, 4% to 53% of patients with GISTs are asymptomatic, found during the endoscopic examination, autopsy, or surgery for other reasons. The diagnostic workup for GIST includes upper gastrointestinal endoscopy and cross-sectional imaging. For operable cases, surgical resection with R0 resection is the standard treatment [3]. The clinical behavior of GIST after surgical resection is extremely variable. Some tumors follow a benign, indolent course, but others show aggressive clinical courses despite adequate oncological resection [2].

Patients with GIST are commonly found to have anemia due to ongoing gastrointestinal bleeding. However, the correlation between tumor volume and the severity of anemia is not established in the available literature. In the present study, we compared the severity of anemia with various factors, mainly tumor volume.

## Materials and methods

This was a retrospective observational study conducted at the Postgraduate Institute of Medical Education and Research, Chandigarh, India. Patients who underwent surgical resection for GIST between July 2018 and March 2021 were included in this study. Ethical approval was given by the Institutional Ethics Committee (INT/IEC/2022/SPL-1365). The data was collected from patient hospital records and entered into an electronic case record form. The data included age, gender, clinical presentation, hemoglobin (g/dl) at presentation, radiological findings, endoscopic mucosal findings (ulceration/normal), tumor location, performed surgical procedure, intra-operative and post-operative complications, and length of stay. The preoperative tumor volume was measured using cross-sectional imaging. The histopathological analysis included tumor characteristics, pathological findings, and immunohistochemical (IHC) analysis. The IHC analysis was done for cKIT and DOG-1. The mitotic rate was measured using high-power fields (HPF). We correlated preoperative hemoglobin levels to preoperative tumor volume, tumor location, and endoscopic mucosal findings.

Statistical analysis

All analyses were two-tailed. The confidence interval was 95%, and p-value <0.05 were considered statistically significant. Categorical variables were analyzed using the chi-square test with Fisher’s exact test and presented as frequencies (percentages). Continuous variables were analyzed using unpaired t-tests and presented as the mean ± SD (standard deviation). Linear correlation between two sets of data is done with Pearson’s correlation coefficient. All the above data analyses were performed using IBM Corp. Released 2016. IBM SPSS Statistics for Windows, Version 24.0. Armonk, NY: IBM Corp.

## Results

A total of 20 patients who underwent surgical resection for GIST were included. All patients underwent upfront surgery. Sixteen patients received adjuvant Imatinib, and four did not receive Imatinib. The mean age of the study group was 53.8 ± 12 years; 11 (55%) were males, and nine (45%) were females. The most common presentation was upper gastrointestinal bleeding (50%, n = 10, melena in five patients, hematemesis in three patients, both in two patients), followed by pain abdomen (35%, n = 7) in one patient presented with lump abdomen, one patient with jaundice, one with dyspepsia. The most common tumor location was the stomach (75%, n = 15), followed by the duodenum (25%, n = 5). In the stomach, the most common location was the body of the stomach (53.3%), followed by the fundus (26.6%) and the antrum (20%). In upper GI endoscopy, 13 (65%) patients have ulcerated mucosa over the tumor; the remaining have normal mucosa. The mean level of hemoglobin in this study was 10.29 ± 1.9 (Table 1). 

**Table 1:** Demographic and descriptive data

Age (Mean ± SD)	53.8 ± 12.4
Male (n, %)	11 (55%)
Symptoms (n, %)	
Upper GI bleed	10 (50%)
Pain abdomen	7, (35%)
Lump	1, (5%)
Jaundice	1, (5%)
Dyspepsia	1, (5%)
Location of tumor (n)	
Body	8
Fundus	4
Antrum	3
Duodenum	5
Overlying Mucosa (n, %)	
Ulcerated	13 (65%)
Normal	7 (35%)
Hemoglobin (Mean ± SD)	10.29 ± 1.9
Tumor Volume (Mean ± SD)	470.8 ± 1269.07
Length of stay (Mean)	13 days
R0 resection (n, %)	18 (90%)
Grade of tumor (n, %)	
Low	10 (50%)
Intermediate	5 (25%)
High	5 (25%)
Immunohistochemistry (n, %)	
c- KIT positive	20 (100%)
DOG positive	11 (55%)

Out of 20 patients, R0 resection was achieved in 18 patients and R1 in two patients. Based on the mitotic activity index on histopathology, 10 patients (50%) had low-grade features, five (25%) had an intermediate grade, and five (25%) had high-grade features. In immunohistochemistry, 20 patients (100%) were c-KIT positive, and 11 patients (55%) were DOG positive, remaining negative for the DOG gene. The average length of stay was 13 days. At two years of follow-up, no patient developed a recurrence.

**Figure 1:**
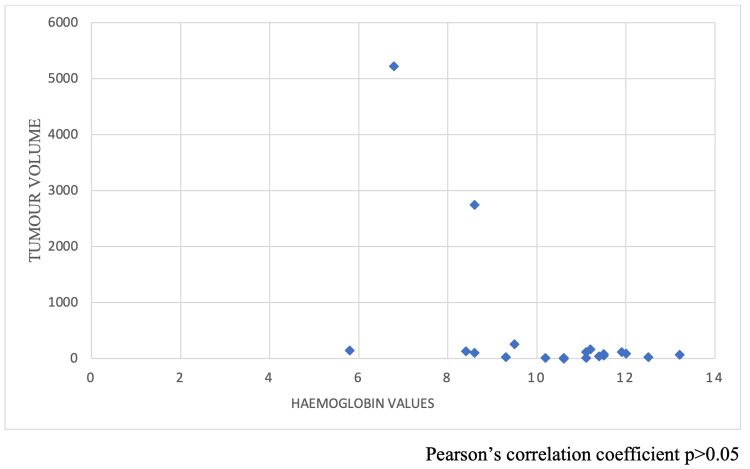
Scattered plot showing the correlation between hemoglobin (g/dL) and tumor volume (CC)

We calculated the tumor volume from the preoperative cross-sectional imaging (length x width x height), then correlated it with hemoglobin levels with Pearson’s correlation coefficient; it showed hemoglobin levels are independent (Figure 1), and there is no correlation with tumor volume. It is also compared with overlying mucosa, histopathological features like the mitotic index, tumor grade, and immunohistochemistry features (c-KIT, DOG), which didn’t show any correlation with hemoglobin levels (p>0.05).

## Discussion

GISTs are highly vascular mesenchymal neoplasms, with the common patient presentation being gastrointestinal bleeding [4]. Anemia in GIST patients is due to both intraluminal and extraluminal bleeding. Among patients presenting to the emergency, tumor rupture and gastrointestinal bleeding are the most common [5]. The severity of anemia in GIST patients has been variable. A large proportion of these patients require a blood transfusion at presentation. Earlier, several studies tried to correlate tumor variables with the severity of anemia. The size of the tumor has been an obvious candidate. Earlier studies of GISTs have compared the tumor size in the largest dimension to look for tumor burden and aggressiveness. This provides a superior measure of the tumor load. Rodeberg et al. [6] showed tumor volume as a stronger predictor of outcomes in children with rhabdomyosarcoma. Till now, there has been no data available for GIST regarding the correlation between tumor volume and its presentation and outcomes. In this study, tumor volume was studied to look for an association with anemia in GIST patients; however, it didn’t show any association.

In the earlier series by Kim et al. [7], the median age of patients with GIST was 60.8 years, with a slight female predominance. However, we observed a median age of 53.8 years with a slight male predominance in this study. Earlier, several studies, including that by Miettinen et al. [8], showed male predominance. The location of gastrointestinal GIST has been described in the stomach in 52%-60% of cases, with 66% of them involving the fundus and body [3,9]. In our series, the stomach was involved in 75% of cases, with 80% of them in the proximal region.

In an Egyptian series, the presentation of GIST was 50% with gastrointestinal bleeding, 30% with intestinal obstruction, 15% with intraperitoneal hemorrhage, and 8% with rupture and peritonitis [10]. Other studies also observed GIST in asymptomatic patients. In our series, all the patients were symptomatic, and gastrointestinal bleeding was the only consistent presentation in 50% of the patients. Other presentations were pain in the abdomen, jaundice, dyspepsia, and a lump in the abdomen. Hence, the presentation of GIST is variable, and gastrointestinal bleeding is the most common and consistent presentation.

In the study by Liu et al. [11], the gastrointestinal bleeding in GIST patients showed a strong association with tumor size, with a cutoff of 5 cm in the single largest dimension. However, the degree of anemia due to it was not studied. Also, the other dimensions or tumor volume were not provided in the study. In our study, the tumor volume of GIST varied between 1 cc and 5225 cc. However, the volume didn’t show a significant correlation with the degree of anemia.

The concept of ‘tumor rupture’, as described by Joensuu et al. [12], has been long hypothesized as a reason for gastrointestinal bleeding in the GIST. The most accepted mechanisms provided in the studies have been mucosal invasion and ulcerations caused by the tumor. However, Miettinen et al. [8] showed that ulcerated mucosa is present in only 39.6% of GIST, and mucosal invasion is rare. In this study, 35% of cases had ulcerated mucosa. The degree of anemia didn’t show a significant correlation with mucosal ulceration. Hence, ulceration does not appear to be the sole reason for anemia in GIST patients.

The location of the tumor has also been correlated with the severity of anemia [11]. The small intestine has been observed as an independent risk factor for gastrointestinal bleeding. Our observations didn’t show any significant correlation between the location and severity of anemia.

In the study by Caterino et al. [3], a mild correlation between symptoms at presentation and the mitotic index was observed (p=0.074). This study identified bleeding and anemia as presenting symptoms in 29.8% of patients. In our study, 50% of patients presented with anemia, but the severity of the anemia didn’t show a significant correlation to the mitotic index of the tumor. Immunohistochemistry has also been studied to find a degree of gastrointestinal bleeding in GIST. The c-kit and DOG-1 have been studied to find an association between these markers and the severity of bleeding. In the same study, CD34 did show a nonsignificant association (p=0.064); however, the authors recommended an additional study to find any significant association. We observed c-kit and DOG-1 in this study, and our findings corroborated the earlier studies.

Our study did have several limitations. This was a single-center retrospective study with a small sample size. The baseline hemoglobin levels of the patients before they were diagnosed with GIST were unknown. The possible cause of anemia due to gastrointestinal bleeding in patients on aspirin was not considered.

## Conclusions

The GIST is a less common mesenchymal gastrointestinal malignancy presenting mostly with anemia. The severity of anemia is not related to tumor size, location, or grade. Mucosal ulceration and gastrointestinal bleeding do not appear to be the sole reason for anemia in GIST patients.

## References

[1] Al-Thani H, El-Menyar A, Rasul KI (2014). Clinical presentation, management and outcomes of gastrointestinal stromal tumors. Int J Surg.

[2] Xu SJ, Zhang SY, Dong LY, Lin GS, Zhou YJ (2021). Dynamic survival analysis of gastrointestinal stromal tumors (GISTs): a 10-year follow-up based on conditional survival. BMC Cancer.

[3] Caterino S, Lorenzon L, Petrucciani N (2011). Gastrointestinal stromal tumors: correlation between symptoms at presentation, tumor location and prognostic factors in 47 consecutive patients. World J Surg Oncol.

[4] von Mehren M, Joensuu H (2018). Gastrointestinal stromal tumors. J Clin Oncol.

[5] Sorour MA, Kassem MI, Ghazal Ael-H, El-Riwini MT, Abu Nasr A (2014). Gastrointestinal stromal tumors (GIST) related emergencies. Int J Surg.

[6] Rodeberg DA, Stoner JA, Garcia-Henriquez N (2011). Tumor volume and patient weight as predictors of outcome in children with intermediate risk rhabdomyosarcoma: a report from the Children's Oncology Group. Cancer.

[7] Kim IH, Kim IH, Kwak SG, Kim SW, Chae HD (2014). Gastrointestinal stromal tumors (GISTs) of the stomach: a multicenter, retrospective study of curatively resected gastric GISTs. Ann Surg Treat Res.

[8] Miettinen M, Sobin LH, Lasota J (2005). Gastrointestinal stromal tumors of the stomach: a clinicopathologic, immunohistochemical, and molecular genetic study of 1765 cases with long-term follow-up. Am J Surg Pathol.

[9] Akahoshi K, Oya M, Koga T, Shiratsuchi Y (2018). Current clinical management of gastrointestinal stromal tumor. World J Gastroenterol.

[10] Menge F, Jakob J, Kasper B, Smakic A, Gaiser T, Hohenberger P (2018). Clinical presentation of gastrointestinal stromal tumors. Visc Med.

[11] Liu Q, Li Y, Dong M, Kong F, Dong Q (2017). Gastrointestinal bleeding is an independent risk factor for poor prognosis in GIST patients. Biomed Res Int.

[12] Joensuu H (2008). Risk stratification of patients diagnosed with gastrointestinal stromal tumor. Hum Pathol.

